# Mortality of the City of Buffalo for the Month of June, 1865

**Published:** 1865-07

**Authors:** 


*Mortality of the City of Buffalo for the Month of June,* 1865.

CAUSES OF DEATH.—Accident 2, Accident, by burn 1, Accident, by drowning 4, apoplexy, cerebral 2, brain, congestion of 1, Bronchitis, 1, Blow on the neck, 1, cancer of the Womb, 3, Cirrohosis of Liver 1, consumption 14, convulsions 8, debility 2, delirium tremens 1, diarrhœa 3, disease of the Liver 1, disease of the spine 1, diphtheria 4, diabetes 1, epilepsy 1, fever, typhoid 2, do. typhus 5, hemorrhage of umbilicus 1, inflammation of bowels 1, do. of brain 1, do. of brain and meningitis 3, do liver 1, do. lungs 5, do. lungs, typhoid 3, do. lungs and pleura 1, insanity 1, meningitis, cere. spin. 2, marasmus 2, Neglect 1, old age 3, perforation of intestine 1, pyæmia 1, rupture of blood vessel 1, scrofula 1, small-pox 11, syphilis 1, ulceration in bowels 1 unknown 1, whooping cough 6. Total 108.

BY WHOM CERTIFIED.—By regular physicians at public institutions 15; do. in city at large 48; by irregular practitioners 20; by coroner 11; by undertakers 22. Total, 116.

